# Practical Considerations for Continuous Time-Domain Cerebrovascular Reactivity Indices in Traumatic Brain Injury: Do Scaling Errors in Parent Signals Matter?

**DOI:** 10.3389/fneur.2022.857617

**Published:** 2022-03-21

**Authors:** Logan Froese, Alwyn Gomez, Amanjyot Singh Sainbhi, Trevor Slack, Frederick A. Zeiler

**Affiliations:** ^1^Department of Biomedical Engineering, Faculty of Engineering, University of Manitoba, Winnipeg, MB, Canada; ^2^Section of Neurosurgery, Department of Surgery, Rady Faculty of Health Sciences, University of Manitoba, Winnipeg, MB, Canada; ^3^Department of Human Anatomy and Cell Science, Rady Faculty of Health Sciences, University of Manitoba, Winnipeg, MB, Canada; ^4^Centre on Aging, University of Manitoba, Winnipeg, MB, Canada; ^5^Division of Anaesthesia, Department of Medicine, Addenbrooke's Hospital, University of Cambridge, Cambridge, United Kingdom

**Keywords:** traumatic brain injury, cerebrovascular reactivity (CVRx), cerebral autoregulation, linearity, continuous monitoring

## Abstract

Literature pertaining to traumatic brain injury care involves the mediation and control of secondary brain injury mechanisms, chief among these is cerebral autoregulation. Cerebral autoregulation is frequently assessed through surrogate measures of cerebrovascular reactivity. An important aspect to acknowledge when calculating cerebrovascular reactivity indices is the linearity within two-parent bio-signals or variables. We highlighted the concept of linearity in raw parent bio-signals used for the calculation of the cerebrovascular reactivity index and what potential implications linearity carries for index derivation. Key of which is that the initial differencing or location of the pressure probes does not influence linear methods of cerebral reactivity calculations so long as the slow-wave vasogenic changes are being recorded.

## Background

Literature pertaining to traumatic brain injury (TBI) care involves the mediation and control of secondary brain injury mechanisms, which are the cascading pathophysiological responses that are incurred due to the primary injury of TBI. The assessment and investigation of these secondary TBI mechanisms may offer potential routes to improve outcomes, which has had little change over the past 25 years (1–4). Among the current secondary mechanisms of injury being investigated, cerebral autoregulation is of note as there is a growing body of literature documenting not only its impact but also various methods of evaluating it at the bedside (5–8).

Cerebral autoregulation is the physiologic mechanism by which homeostatic cerebral blood flow (CBF) is maintained (5, 9, 10). Frequently, it is assessed through surrogate measures of cerebrovascular reactivity (6–8, 11–15). Such assessment of cerebrovascular reactivity has shown that the current Brain Trauma Foundation guideline-based management has had a limited impact on cerebral autoregulatory dysfunction following TBI (1, 16, 17). This has sparked interest in the field of neurocritical care as an improved understanding of dysfunctional autoregulation may provide a potential route for improved patient outcomes (12, 16, 17). This enthusiasm has been bolstered by evidence indicating that dysfunctional autoregulation is linked to poor outcomes following TBI (8, 12, 17, 18).

Contemporary methods of continuously measuring cerebrovascular reactivity involve the comparison of systemic blood pressure/flow to an aspect of cerebral response associated with vascular control. These methods focus on the slow wave vasogenic fluctuations that are assumed to be associated with cerebrovascular reactivity, mostly within ~0.005–0.5 Hz, though this is still underexamined (7, 13, 19, 20). The most commonly utilized method to assess cerebrovascular reactivity is the pressure reactivity index (PRx), which correlates the mean arterial pressure (MAP) and the intracranial pressure (ICP) (6, 8, 13). Usually, systemic blood pressure is extracted in the form of arterial blood pressure (ABP) with ICP being extracted from an intraparenchymal strain gauge probe. Both the MAP and the ICP are derived for the grand average mean over a 10-s window of ABP and ICP, respectively. Next, the Pearson's correlation between the MAP and ICP for 30 consecutive 10-s windows is derived, thus comparing the slow-wave vasogenic relationship between MAP and ICP. The 10-s window is respective of the 0.1 Hz associated with the upper limit of the slow-wave vasogenic response.

## Point of Common Concern

An important aspect to acknowledge when calculating cerebrovascular reactivity indices is the linearity within two-parent bio-signals or variables. Specifically, a common concern is raised regarding the introduction of scaling “errors” to the parent bio-signals that are used in the calculation of time-domain-based cerebrovascular reactivity indices, such as PRx. These scaling “errors” can take the form of zero errors, for example, where the ABP transducer is zeroed at the right atrium vs. level of the tragus. We highlighted below the concept of scaling errors potentially introduced to the raw parent bio-signals used for cerebrovascular reactivity index calculation, and what potential implications those carry for index derivation.

## Impact of Scaling Errors in Parent Bio-Signals on Cerebrovascular Reactivity Index Derivation

**Figure 1:**
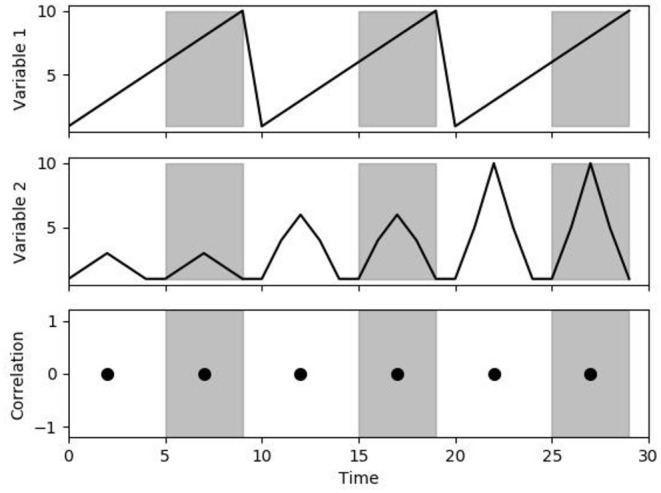
Correlation as a change in multiplicative scaling. The figure demonstrates the correlation between variables 1 and 2 over a 5-s time window. Note that despite the change in scaling, the correlation is always 0 (linearity is preserved).

**Figure 2:**
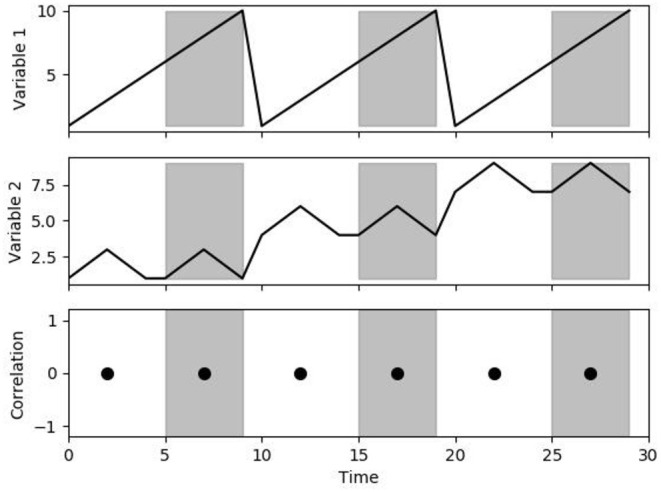
Correlation to additive changes. The figure demonstrates the correlation between variables 1 and 2 over a 5-s time window. Note that despite the change in mean value, the correlation is still 0 (linearity is preserved).

Linearity refers to a transformation that satisfies two aspects; additivity and scaling property (21). The additive property states that if the same amount is added to both sides of an equation, then the equality is still true (addition or subtraction by a constant). The scaling property states that an equation can be multiplied by a positive real number, which only changes the magnitude of response (multiply or divide by a positive number). By maintaining linearity, the transformation to a variable can be caused by the sum of two or more stimuli, which would be equal to the response of each stimulus individually at the same time. Examples of a linear modification would be any simple arithmetic changes to the data by a vector (plus/minus and multiply by a positive number). As long as linearity is preserved, linear techniques (like Pearson's Correlation = $\frac{\sum \left(x_{i\;}+\;\bar{x}\right)\left(y_{i\;}+\;\bar{y}\right)}{{\sum \left(x_{i\;}+\;\bar{x}\right)}^{2}{\sum \left(y_{i\;}+\;\bar{y}\right)}^{2}}\;$) are equivalent between the data (see Figures 1, 2).

From Figures 1, 2 it can be seen that modifications to the original signal's waveform that maintain linearity would demonstrate identical results in linear correlations. For PRx, both MAP and ICP can have a wide variation in mean value over the whole data or a significant variation in the magnitude of patient response. The importance of linearity means that the location of the zeroing transducer for ABP monitoring does not influence the calculation of PRx. Similarly, for the ICP waveform, as long as the slow-wave vasogenic changes in ICP are being recorded, cerebrovascular reactivity can be determined. Ultimately, cerebrovascular reactivity as determined through PRx is the relationship of change between the MAP and ICP, thus the overall response factors, like multiplicative scaling, do not influence the resulting PRx value. Likewise, the PRx calculation is indeterminate of the initial ICP and MAP placement, and thus the zeroing of the MAP at the right atrium vs. level of the tragus have an identical resulting PRx. Furthermore, within ICP monitoring, there is a trend for the mean value to drift very slowly over the course of the recording period. If the change is sufficiently slow in relation to the frequency at which PRx is being calculated, this drifting of ICP would have a negligible impact on PRx (i.e., if ICP drifts 1–2 mmHg over a day and PRx is calculated every 5 min).

## Impact on “Other” Multi-Modal Cerebrovascular Reactivity Indices

Other multi-modal cerebral physiologic monitoring has been employed for the derivation of alternative cerebrovascular reactivity indices in moderate/severe TBI. These are derived using the same principle as outlined above for PRx derivation. Such monitoring devices utilized include the following: brain tissue oxygenation as collected through a probe that measures oxygen tension, near-IR spectroscopy to detect regional cerebral oxygen saturation, transcranial doppler to measure CBF velocity, and thermal diffusion-based CBF as measured through parenchymal probes (22). The literature on the utility of such cerebrovascular reactivity indices in TBI care remains limited. Regardless, scaling errors introduced into these parent cerebral physiologic signals derived from various multi-modal monitors lead to the same mathematical considerations raised in the section above on PRx derivation. That is, the introduction of constant additive or multiplicative scaling error into these parent signals carries no impact on the derivation of Pearson's correlation coefficient that forms these alternative cerebrovascular reactivity indices. Similarly, wherever linear correlation coefficient values are being derived, a linear modification to the original data would not influence the calculated correlation coefficient.

## Conclusions

In conclusion, cerebral autoregulation offers a potential avenue to better improve patient care. In line with this, cerebrovascular reactivity offers a potential way to assess autoregulation in a patient. This is done often through linear equations, which means that, as long as linearity is preserved in the acquired data, the calculation of cerebrovascular reactivity indices will remain accurate and mathematically independent of any scaling “error” applied to the parent signals.

## Data Availability Statement

The original contributions presented in the study are included in the article/supplementary material, further inquiries can be directed to the corresponding author.

## Author Contributions

LF, AG, and FAZ contributed to conception and design of the study. LF wrote the first draft of the manuscript. All authors contributed to manuscript revision, read, and approved the submitted version.

## Funding

This study was supported by the Manitoba Public Insurance (MPI) Neuroscience/TBI Research Endowment, the Health Sciences Centre Foundation Winnipeg, and the University of Manitoba Department of Surgery GFT Research Grant program. FAZ received research support from the Manitoba Public Insurance (MPI) Neuroscience/TBI Research Endowment, the Health Sciences Centre Foundation Winnipeg, the United States Nationals Institutes of Health (NIH) through the National Institute of Neurological Disorders and Stroke (NINDS) (Grant #: R03NS114335-01), the Canada Foundation for Innovation (CFI) (Project #: 38583), Research Manitoba (Grant #: 3906), the University of Manitoba VPRI Research Investment Fund (RIF), the University of Manitoba Centre on Aging, and the University of Manitoba Rudy Falk Clinician-Scientist Professorship. AG was supported through the University of Manitoba Clinician Investigator Program, the University of Manitoba Dean's Fellowship, the Manitoba Medical Services Foundation Research and Education Fellowship, and the R. Samuel McLaughlin Research Fellowship. AS was supported through the University of Manitoba, Department of Surgery GFT Research Grant. LF was supported through the University of Manitoba, Department of Surgery GFT Research Grant and the University of Manitoba Office of Research Services (ORS) – University Research Grant Program (URGP) and Biomedical Engineering Fellowship.

## Conflict of Interest

The authors declare that the research was conducted in the absence of any commercial or financial relationships that could be construed as a potential conflict of interest.

## Publisher's Note

All claims expressed in this article are solely those of the authors and do not necessarily represent those of their affiliated organizations, or those of the publisher, the editors and the reviewers. Any product that may be evaluated in this article, or claim that may be made by its manufacturer, is not guaranteed or endorsed by the publisher.
